# Changes in Glenohumeral Musculoskeletal Development Following Brachial Plexus Birth Injury

**DOI:** 10.1002/jor.26104

**Published:** 2025-06-08

**Authors:** Emily B. Fawcett, Kyla B. Bosh, Katherine R. Saul, Jacqueline H. Cole

**Affiliations:** ^1^ Joint Department of Biomedical Engineering University of North Carolina, Chapel Hill, NC, and North Carolina State University Raleigh North Carolina USA; ^2^ Mechanical and Aerospace Engineering North Carolina State University Raleigh North Carolina USA

**Keywords:** bone, brachial plexus, muscle, pediatric, shoulder

## Abstract

Brachial plexus birth injury (BPBI), one of the most common nerve injuries in children, often leads to impaired shoulder development, resulting in sustained postural and bone deformity and muscle weakness. Despite the substantial long‐term consequences, clinical consensus is lacking for what BPBI treatments are optimal in terms of timing and approach, primarily because BPBI sequelae are complex, involving stunted muscle growth, muscle denervation, and limb disuse that can disrupt glenohumeral joint development. Injury can occur as nerve rupture (postganglionic injury) or nerve avulsion (preganglionic injury), which have distinct musculoskeletal consequences yet are often treated similarly clinically due to their similar initial presentations and the inability of existing methods to distinguish between them. Most of our clinical knowledge about the musculoskeletal detriments in the shoulder comes from studies in nerve rupture patients. Knowledge is generally lacking for the specific effects of injury location on the development and progression of muscle and bone deficits following BPBI. A better understanding of the distinct effects of postganglionic and preganglionic BPBI is important for developing more effective and targeted treatments. More studies are needed to elucidate differences between nerve rupture and nerve avulsion and the particular factors driving glenohumeral deformity development. This paper reviews current knowledge about clinical musculoskeletal deformity development in the shoulder following BPBI, as well as additional insights gleaned from animal and computational models, and identifies key gaps that need to be addressed in future studies to inform better approaches for mitigating and preventing glenohumeral deformity in these patients.

## 1 Introduction

Brachial plexus birth injury (BPBI) occurs during a difficult childbirth when the neonate's head and shoulder experience traction, excessively stretching and damaging the brachial plexus innervating the shoulder and arm from the C5‐T1 vertebrae. One of the most common pediatric nerve injuries, BPBI occurs in about 0.9 out of every 1,000 births [1], resulting in lifelong arm impairment in 30–40% of those affected [2]. BPBI risk factors include shoulder dystocia [3], which increases injury likelihood by 10.3% [4]; gestational diabetes, which has been associated with higher birth weights [5]; and uterine tachysystole, or excessive contractions [6]. While causal birth factors are understood, and injury incidence has gradually decreased from 1.2 out of 1,000 births since 1997 [1], little focus has been applied to factors of postnatal injury development and progression. Understanding key drivers of postnatal injury progression can lead to better treatments for mitigating or preventing life‐altering deformities occurring post‐injury.

Despite potentially severe long‐term consequences with BPBI, clinical consensus is lacking for optimal treatment timing and approach [7, 8] because of the complexity of postnatal musculoskeletal development. BPBI‐related deformities present differently than deformities with adult peripheral nerve injury [9], and so the abundance of research in adult injuries cannot be directly applied. Neonates have an immature musculoskeletal system that undergoes rapid development with continual formation of new bone at the growth plates [9]. Alterations to bone metabolism disrupt normal musculoskeletal development, and disruptions in muscle growth alter mechanical loading on bone, which affects bone mineralization and growth [10, 11, 12, 13]. Though consequences of peripheral nerve injury affect the musculoskeletal system, they are not commonly investigated and characterized in the pediatric population.

Treatment plans in young children with BPBI have not been as comprehensively studied as in adults with brachial plexus injuries [7], especially when it comes to timing of surgical intervention [4]. Neonates with BPBI remain in the hospital approximately 20% longer than healthy neonates and have significantly higher hospital costs [4, 14]. This additional monitoring has not been found to affect recovery rates [4], likely due to lack of knowledge surrounding deformity development and prevention strategies. After hospital discharge, typical treatments to mitigate altered joint morphology post‐injury include physical therapy, surgery, or waiting for spontaneous nerve recovery [15, 16].

Physical therapy primarily focuses on increasing muscle passive range of motion to prevent joint contracture [17] but may ignore other detriments including altered bone morphology. Electrical stimulation can also be used to improve muscle function and strength in BPBI patients, though it is not part of standard care and no standardized protocols exist for timing [18] and specific usage parameters [19]. A few studies of electrical stimulation used in conjunction with range of motion therapies and surgeries following BPBI have reported improved joint function with increased active range of motion for shoulder abduction, shoulder flexion, elbow flexion, and wrist extension [18, 20, 21, 22]. One study also reported increased humeral bone mineral density in BPBI subjects with electrical stimulation used in conjunction with weight‐bearing exercises [20].

Surgery focuses on nerve grafts to replace injured nerve tissue, which is frequently used with secondary tendon transfer surgeries for long‐term success [17], or nerve transfer to correct innervation of shoulder external rotator or abductor muscles. Few surgeries successfully reinnervate and repair altered glenohumeral joint morphology simultaneously [16], potentially due to controversy in surgery timing and types. In some cases, nerve reconstruction procedures worsened osseous deformity, specifically posterior subluxation, glenoid version, and scapular elevation, often resulting in secondary operations [23].

Some clinicians delay physical therapy and attempt to avoid surgery by implementing a “wait‐and‐see” approach to allow for potential spontaneous nerve regeneration. During this period, rapid musculoskeletal growth occurs, deformities develop, and damage becomes irreversible. If treatment plans have not induced substantial recovery by 3 months after birth, deficits become permanent, including limited joint range of motion, decreased muscle strength, and decreased limb length and girth [24]. Understanding what deformities are occurring, and when and how they develop, are essential for determining optimal treatment type and timing. This review summarizes current knowledge regarding musculoskeletal deformity formation following BPBI to inspire new scientific studies and ultimately help inform more effective treatment strategies for those with pediatric peripheral nerve injuries.

### 1.1 Injury Type and Diagnosis

**Figure 1:**
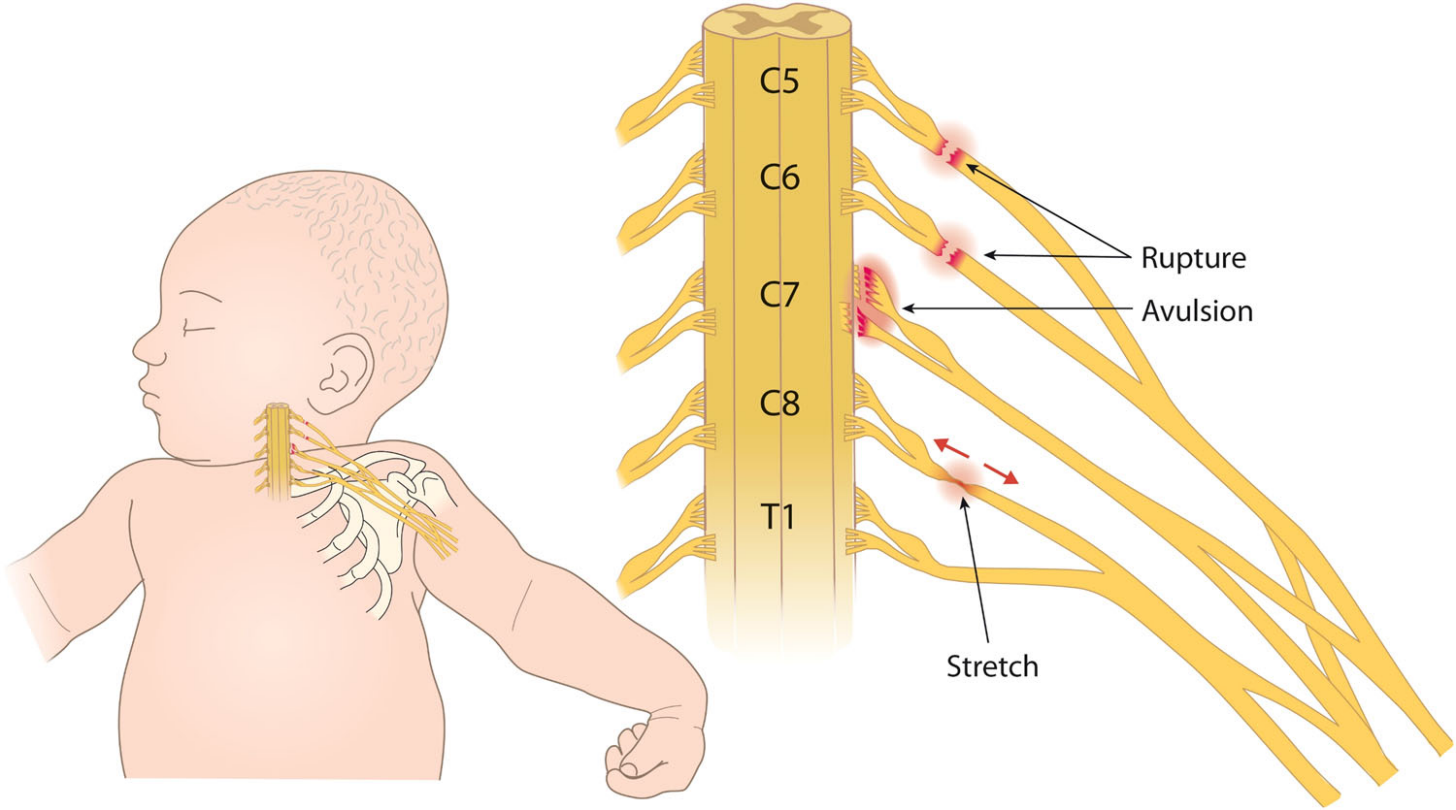
Anatomy of the brachial plexus nerve bundle from the C5‐T1 nerve roots. Nerve rupture occurs distal to the dorsal root ganglion (*postganglionic injury*). Nerve avulsion occurs proximal to the dorsal root ganglion (*preganglionic injury*). Image adapted and used with permission [33].

Following BPBI, children develop postural and osseous deformities that worsen with age [25, 26]. Common co‐morbidities contributing to deformities are joint contracture and limb disuse, though severity of these co‐morbidities depends on injury type/location [27]. Patients with nerve rupture, occurring distal to the dorsal root ganglion, experience joint contracture [25, 28, 29] and limb disuse [25], while patients with nerve avulsion, occurring proximal to the dorsal root ganglion where afferent sensory innervation is potentially preserved, experience limb disuse [30] without joint contracture [31, 32] (Figure 1 [33]). While these two injury types damage the brachial plexus network in different ways, the initial clinical presentation is similar [2], which makes distinguishing between them difficult and results in similar treatments. By 2 weeks of age, BPBI patients should be classified by the Narakas classification system (Table 1) to aid in injury prognosis, with the likelihood for spontaneous recovery decreasing from Group 1 to Group 4 [34]. While Narakas classification does not specifically distinguish between nerve rupture and nerve avulsion, nerve avulsion can be inferred with the presence of Horner's syndrome [16], though characterization requires additional examinations through magnetic resonance imaging [35, 36, 37, 38], radiography [39], computed tomography [40], or ultrasound [41, 42].

**Table 1:** Narakas Classification System [34].

Classification & label	Nerve roots involved	Affected limb presentation
Group 1: Upper plexus	C5‐C6	Elbow flexion & shoulder external rotation
Group 2: Extended upper plexus	C5‐C7	Drop wrist, elbow flexion, & shoulder external rotation
Group 3: Total palsy without Horner's syndrome	C5‐T1	Total limp limb paralysis
Group 4: Total palsy with Horner's syndrome	C5‐T1	Total limp limb paralysis with Horner's syndrome

The distinction of injury type (nerve rupture vs. avulsion) is not frequently described in clinical studies beyond the location of affected nerve roots (e.g., C5‐C6), and so their prevalence is not well reported. However, nerve avulsions tend to occur more often in lower nerve roots [39, 40, 43, 44]. In addition, several diagnostic imaging studies have distinguished nerve rupture and nerve avulsion when validating with surgical exploration before surgery. One study with 25 upper brachial plexus explorations reported that 33.8% of the total lesions found were avulsions (22 avulsions, 43 ruptures) [43]. Other studies reported the presence of avulsions relative to the total number of nerve roots examined, with 13.5% avulsions (38 out of 281 at C5‐T1) for 58 BPBI patients [44], 18.1% avulsions (19 out of 105 at C5‐T1) for 21 BPBI patients [40], 32.1% avulsions (135 out of 420 for C4‐T1) for 52 BPBI patients [39], and 42.1% avulsions (8 out of 19 at C5‐T1) for 6 BPBI patients [45]. One study noted the prevalence of nerve root avulsions in 52 out of 70 BPBI patients (74.3%) [39]. These studies show that, while the reported prevalence of nerve root avulsions within the brachial plexus is wide‐ranging, avulsions are not a rare occurrence clinically and remain understudied relative to the more in‐depth knowledge of nerve root ruptures in the brachial plexus.

Overall, little is understood about the role that injury type/location plays in the development and progression of musculoskeletal deformities and deficits following BPBI.

## 2 Clinical Studies

### 2.1 Bone Deformities

Following BPBI, gross alterations to bone morphology have been observed throughout the glenohumeral joint, occurring to both the humeral head and glenoid region of the scapula. These morphological changes present as early as 3 months old [26, 36, 46, 47, 48] and continually worsen with development [25, 26, 49]. Most clinical studies do not differentiate between nerve rupture and nerve avulsion. Following unspecified BPBI, the humerus is posteriorly subluxated [25, 26, 29, 50, 51], and the humeral head is smaller [29] and flattened [51, 52]. A few studies have reported delayed ossification [53], decreased humeral length [54], and either a retroverted [55] or anteverted [56] humerus. Common scapular changes include subluxation [23, 47], retroversion [25, 26, 29, 50, 57], and elevation [58, 59], with some studies also reporting scapulae that are convex [26, 57] or biconcave [26]. In the presence of joint contracture, which follows only after nerve rupture [32], osseous deformity increases with increasing contracture severity [60]. Studies suggest that overall scapular deformity [15] and flattening of the humeral head [52] are associated with, and indicators of, joint contracture.

### 2.2 Muscle Detriments

Although a common early treatment for BPBI is physical therapy to stretch muscles [15], few clinical studies investigate alterations in muscle morphology, especially regarding injury location. Clinical studies that did examine muscles surrounding the glenohumeral joint reported decreased muscle size and volume [47, 54, 61, 62] and increased atrophy [35, 36, 47] and stiffening [61], though none of these studies specified nerve rupture or avulsion. Muscles most severely affected by BPBI across all studies were the subscapularis [35, 47, 50, 62] and deltoid [35, 47]. Atrophy of these muscles was significantly associated with more severe glenohumeral joint contracture [35]. Since contracture has previously been reported only after nerve rupture, the correlation between muscle atrophy and contracture is assumed to be in the context of nerve rupture.

### 2.3 Clinical Correlations

In clinical BPBI studies, correlations have been observed between various bone and muscle metrics. Increased posterior subluxation of the humerus relative to the scapula is associated with overall worsened glenoid deformity [47], decreased glenoid version [25, 29, 47], decreased scapular length [58, 63], and decreased passive range of motion [47, 64, 65]. Both scapular and humeral deformities have been associated with muscle detriments, with the scapula more tightly correlated to muscle alterations. Joint subluxation is related to decreased infraspinatus and subscapularis muscle volume [47] and subscapularis atrophy [35, 50]. Glenoid version angle has been correlated with subscapularis atrophy and fatty degeneration [50] and other rotator cuff muscle atrophy [35]. Considering these observations, scapular deformity seems to be most associated with worsened architecture and atrophy of the subscapularis muscle, followed closely by the infraspinatus; and both scapular deformity and joint subluxation are related to muscle alterations affecting mechanical loading of the glenohumeral joint. These studies did not differentiate between injury types and likely examined only nerve rupture due to contracture presence.

### 2.4 Significance of Clinical Findings

While musculoskeletal deformities of the shoulder are well characterized following BPBI, our clinical knowledge has been primarily derived from nerve rupture patients. Since distinguishing between injury types remains difficult during early stages, prognoses for deformity development cannot be accurately predicted in individual cases. Understanding more about differences between nerve ruptures and nerve avulsions and what factors drive deformity development is important for creating more effective and targeted treatments. These factors cannot be explored in clinical populations due to the inability to manipulate and control the drivers. Animal models are critical for advancing our understanding in this area and establishing predictive models for deformity development and progression.

## 3 Animal Studies

Murine models have been developed to mimic both postganglionic (nerve rupture) and preganglionic (nerve avulsion) injuries, where surgical denervation with a C5–C6 nerve root excision is performed at 3–5 days following birth. While neurectomy procedures differ from how injuries are incurred at birth, they recapitulate glenohumeral joint abnormalities seen clinically [66, 67, 68, 69, 70], as described in subsequent sections below. In postganglionic neurectomy the nerve root is excised *distal* to the dorsal root ganglion, mimicking clinical nerve rupture and leading to severe joint contracture and bone deformity [66, 68], with restricted longitudinal muscle growth and degenerative changes to muscle spindles [71, 72, 73]. In preganglionic neurectomy the nerve root is excised *proximal* to the dorsal root ganglion, mimicking clinical nerve avulsion and, compared to postganglionic neurectomy, leading to markedly less joint contracture [71] and bone deformity [67] and shorter, more atrophic muscles [74]. Many animal studies either do not specify injury type or only use the postganglionic injury model; few studies have compared preganglionic and postganglionic BPBI, despite clinical evidence suggesting they have functional and physiological differences related to joint contracture and glenohumeral deformity. Future studies should use both models to compare how deformities develop and persist to advance our understanding of underlying differences between injury types, which could lead to more targeted treatments and better functional outcomes clinically.

### 3.1 Bone Macrostructure

Glenohumeral deformities at the macrostructural level have been thoroughly investigated in murine models of postganglionic BPBI, and results mirror clinical observations. In these studies, the glenoid exhibited extensive changes in joint positioning and morphology following BPBI, becoming retroverted [69], declined [68], and flatter in curvature [67]. Humeral length was shorter [69, 75, 76], and the humeral head was smaller [67], less retroverted, and flattened [67, 69, 75] with BPBI. These murine BPBI models showed greater macrostructural changes in the scapula than in the humerus [67, 68, 77], similar to clinical observations.

A couple of previous studies in rat models examined different injury types and reported distinct differences in glenohumeral deformity between postganglionic and preganglionic injuries [67, 77]. Following postganglionic injury, only glenoid deformities were present, with no humeral head deformities observed. The injured glenoid was more declined and flatter in curvature. Following preganglionic injury, the humeral head width, length, and curvature were decreased [67], suggesting reduced bone growth. Given these differences in glenohumeral deformities between injury types, additional research is needed to better understand different injury sequelae and underlying factors driving them so that clinical treatments and therapies can be developed to target specific injury types.

### 3.2 Bone Microstructure

While trabecular bone architecture is not currently investigated in clinical BPBI populations, it plays an important role in load‐bearing and overall biomechanics of the shoulder, and some studies have examined microstructural bone changes in murine models. Some studies only looked at postganglionic injury and analyzed the humeral epiphysis [78, 79]. One study assessed postganglionic injury relative to a sham surgical control and analyzed the humeral epiphysis, humeral metaphysis, and three scapular regions [80]. In these studies, humeral trabecular architecture was declined following BPBI, with decreased trabecular number and thickness and greater trabecular separation [78, 80]. Additionally, bone mineral density (BMD) was reduced in the humeral epiphysis but remained unaffected in the humeral metaphysis [79, 80]. Microstructural bone detriments with BPBI were greater in the scapula than in the humerus and were most pronounced in the neck region near the glenoid surface, with large deficits in BMD and trabecular number [80].

Overall, these results revealed microstructural, but not macrostructural, changes in the humeral head following postganglionic BPBI, suggesting that driving factors for bone changes in the humerus with this injury type may be different for joint morphology and trabecular architecture. The scapular results revealed stark, microstructural *and* macrostructural detriments following postganglionic BPBI, suggesting that either disruptions in glenoid trabecular architecture may contribute to more severe morphological deformities of the scapula or that driving factors may be concurrently contributing to both. Additional research is needed, both clinically and in animal models, to elucidate distinct musculoskeletal effects with the injury types, understand any relationships and common drivers between microstructural and macrostructural changes, and reveal mechanisms underlying deformity.

### 3.3 Muscle Alterations

Muscle architecture has been characterized in murine BPBI models for muscles surrounding the shoulder and elbow. Postganglionic neurectomy stunted longitudinal muscle growth, with decreased muscle length [72, 73, 74], volume [71, 76, 78], mass [74], and cross‐sectional area [70, 71, 72, 73], and increased sarcomere length [71, 73], though these effects varied across specific muscles. Muscle volume was decreased in the biceps [71], brachialis [71], and supraspinatus [76, 78], and cross‐sectional area was decreased in the biceps and brachialis [71, 72, 73]. Muscle mass was reduced in the pectoralis major, anterior deltoid, spinodeltoid, biceps long head, subscapularis, teres major, and triceps [74]. Optimal muscle length was shortened in the pectoralis major, spinodeltoid, and subscapularis [64], and lengthened in the biceps long head [74]. Sarcomere length was increased in the brachialis [73], subscapularis [73], and teres major [74].

Muscle architecture changes following preganglionic injury have only been characterized in a few studies, with one study reporting substantially decreased muscle mass and optimal muscle length and increased sarcomere length, as with postganglionic injury [74]. The effect of preganglionic injury on other architectural metrics has not been reported. For specific muscles, muscle mass was reduced in the anterior deltoid, spinodeltoid, biceps long head, subscapularis, supraspinatus, infraspinatus, teres major, and triceps [74]. Optimal muscle length was reduced in the spinodeltoid, biceps long head, biceps short head, supraspinatus, and teres major [74], and sarcomere length was increased in the pectoralis major, biceps long head, biceps short head, and teres major [74].

Comparing injury types, worse detriments in muscle architecture were observed after preganglionic injury than after postganglionic injury [74]. Preganglionic injury induced greater decreases in muscle mass and optimal muscle length compared to postganglionic injury, and it also affected more individual muscles [74]. Decreases in muscle mass were significantly greater for preganglionic than postganglionic injury in the anterior deltoid, subscapularis, supraspinatus, and infraspinatus, and decreases in optimal muscle length were greater in the biceps long head, biceps short head, and supraspinatus [74]. Overall, postganglionic and preganglionic injuries both produced shorter and smaller muscles, with preganglionic injury having a more drastic effect.

A few studies have characterized the effects of BPBI on muscle composition, satellite cells, and muscle spindles. Muscle fibrosis was observed in the biceps [71, 72, 73] following both postganglionic [71, 72, 73, 81] and preganglionic [71, 81] injury and to a lesser extent in the brachialis [71, 72, 73] following postganglionic injury (not examined with preganglionic injury) [72] and the subscapularis [61, 62] following preganglionic but not postganglionic injury [81]. Postganglionic injury resulted in fatty infiltration in the supraspinatus [73, 76] and an accumulation of satellite cells in all stages of activation, proliferation, differentiation, and myotube formation [72]. These metrics have not been examined with preganglionic injury. One research group investigated muscle spindles and ErbB signaling, which is involved in myogenesis and muscle regeneration and is important for recovery following nerve injury [71, 82]. In postganglionic injury, the muscle spindle intrafusal fibers and surrounding capsule were degenerated, and ErbB signaling from the spindles was disrupted. However, in preganglionic injury the spindles and their ErbB signaling were preserved, likely due to the partial preservation of afferent signaling. Both postganglionic and preganglionic injuries caused upregulated ErbB signaling from denervated extrafusal fibers. Overall, these studies suggest that muscle composition becomes more fibrotic after both injury types, and muscle spindle structure and function are degenerated after postganglionic injury. Because muscle spindles are mechanoreceptors, detriments to them likely contribute to impaired muscle growth following postganglionic BPBI [71].

Although more muscle metrics have been examined in animal studies, the results generally align with those seen clinically, especially with postganglionic injury and nerve rupture. More studies are needed to improve our understanding of how injury type affects muscle structure and function, and how alterations in muscle and bone interact. Though animal models have revealed some compositional and cell‐signaling detriments after injury in certain muscles, we do not yet understand the effects on other muscles surrounding the shoulder and elbow or, more importantly, the underlying mechanisms driving these changes. Improving knowledge of the underlying mechanisms driving muscle changes, and how muscle detriments are associated with osseous deformities, are crucial first steps for constructing enhanced treatment plans to mitigate or prevent deformities.

### 3.4 Relationship to Contracture

Reduced longitudinal muscle growth, specifically in the biceps, brachialis, and subscapularis [70, 72, 73, 74, 82], has been implicated as a primary contributor to contracture severity. The brachialis muscle seems to contribute most, as elbow joint contracture is lessened when this muscle is removed [72, 73]. However, muscle shortening is not likely the sole factor behind specific joint contracture [71, 72, 82], since optimal muscle length is significantly shortened even without contractures after preganglionic injury [73, 74]. The smaller muscle mass after preganglionic injury is protective against contracture, since it limits passive force production [73, 74]. Contracture is also not likely related to fibrosis development, as elbow flexion contractures occurred as early as 2 weeks after injury in a mouse model of postganglionic BPBI [73, 76], while fibrosis did not develop until 4 weeks after injury, and fibrosis was not correlated with the degree of contracture at 4 weeks [73]. Lastly, denervation itself likely does not cause contracture, since muscle denervation is similar between postganglionic and preganglionic injury, though this has only been assessed in the biceps brachii muscles [71, 82] and needs to be expanded to other muscles impacted by BPBI.

## 4 Loading Alterations

Studies have also investigated to what extent altered forms of joint loading may be playing a role in BPBI‐related macrostructural bone deformities. Mechanical loading of bone is critical for proper growth and development [83, 84]. Bone provides structural support and adapts to the load it experiences [83, 84, 85]. Altered joint loading following BPBI could contribute to osseous deformities through strength imbalance, restricted longitudinal muscle growth, and/or limb disuse. Strength imbalance has been investigated by injecting botulinum neurotoxin A (Botox) into the supraspinatus muscle [86, 87, 88] or the posterior muscles of the shoulder joint [68], causing muscle imbalances seen with BPBI but with nerves intact. Restricted muscle growth has been assessed using a combination of neurectomy that denervates external rotator muscles [72] plus Botox injected into the anterior muscles of the shoulder to reduce the strength imbalance between internal and external rotators [68]. Limb disuse has been examined through forearm amputation to represent altered usage of the limb, with elbow walking as seen in BPBI [88] but without nerve injury [89].

### 4.1 Strength Imbalance

Studies using strength imbalance models have assessed macrostructural osseous changes to the glenohumeral joint and architecture changes in muscles surrounding the shoulder. When the supraspinatus was paralyzed with Botox, shoulder range of motion was reduced and glenohumeral joint deformities were prevalent [86], including substantially smaller, flattened, and anteverted humeri and smaller and retroverted scapulae, similar to postganglionic BPBI. With Botox‐induced paralysis of either the supraspinatus or posterior shoulder muscles, muscle architectural changes similar to preganglionic BPBI were observed, including greater shoulder range of motion, decreased muscle mass in supraspinatus, infraspinatus, and spinodeltoid muscles, and shortened optimal muscle length in spinodeltoid and teres major [68, 86]. These studies also observed decreases in muscle volume and strength and higher amounts of fibrosis, muscle atrophy, and fat accumulation that increased over time following Botox injections [68, 86, 87], which is similar to postganglionic and preganglionic BPBI.

### 4.2 Restricted Muscle Growth

The study using the restricted muscle growth model assessed changes in muscle mass and length with respect to glenohumeral joint deformity [68]. With combined paralysis of external rotator muscles (via neurectomy) and internal rotator muscles (via Botox), shoulder external rotation range of motion was reduced. In this study, restricted muscle growth resulted in more severe shoulder deformity than did strength imbalance, including glenoid declination and inferior humeral head translation. Combined paralysis of external and internal rotator muscles resulted in decreased muscle mass in biceps and triceps and shorter optimal muscle lengths for some internal rotator muscles (pectoralis major, subscapularis, and teres major), and supraspinatus [68]. The observed morphologic alterations in both bone and muscle are similar to what is observed following postganglionic BPBI.

### 4.3 Computational Models

Computational models have been used to understand the separate contributions of strength imbalance and restricted muscle growth to postural and osseous shoulder deformity with BPBI [90, 91, 92]. In strength imbalance models, regarding postural deformity, the subscapularis, anterior deltoid, and pectoralis major muscles contributed most to reduced external shoulder range of motion [91]. Muscles observed to be most mechanically capable of contributing to osseous deformity via compressive forces were the infraspinatus, latissimus dorsi, and subscapularis [91]. These data suggest strength imbalance is a driving factor after both postganglionic BPBI, based on scapular macrostructural deformities, and preganglionic BPBI, based on changes in muscle architecture and composition. In restricted muscle growth models, regarding postural deformity, the anterior deltoid, subscapularis, and triceps long head affected external shoulder range of motion [91]. Muscles observed to have the greatest contribution to osseous deformity were infraspinatus, subscapularis, triceps long head, and biceps long head [91]. These data suggest that altered muscle forces due to restricted muscle growth contribute to osseous deformity following postganglionic BPBI. Overall, these studies indicate that with both strength imbalance and restricted muscle growth the subscapularis muscle is a main contributor to both postural and osseous deformities, while the infraspinatus likely also contributes to osseous deformity.

### 4.4 Limb Disuse

Studies using forearm amputation models for limb disuse have assessed changes to shoulder macrostructural deformities and microstructural osseous detriments [77] and alterations to muscle architecture [89]. With limb disuse the glenoid curvature was flatter, the glenoid inclination angle was smaller, and the humeral head was smaller, similar to what is observed following postganglionic BPBI [77]. The microstructural bone changes were also similar to what is observed following postganglionic BPBI, most notably less bone quantity, and sparse but thicker trabeculae [77]. Muscle architectural changes included decreased mass in the biceps long head and triceps long head, decreased sarcomere length in the acromiodeltoid and subscapularis, increased sarcomere length in the spinodeltoid and teres major, and a decrease in optimal muscle length in the biceps long head, biceps short head, and triceps long head but no observed differences in levels of muscle fibrosis [89]. A computational study modeling limb paralysis showed that static loading, which is associated with paralysis and disuse, produces glenoid declination and flattening similar to postganglionic BPBI, though the combination of static loading and restricted muscle growth recapitulated the extent of altered joint morphology more closely [90]. Although strength imbalance, restricted longitudinal muscle growth, and limb disuse are all associated with altered muscle structure, we cannot infer which one has the greatest effect on muscle alterations, since different metrics were measured in the three studies, and studies comparing these conditions are lacking.

These studies suggest the musculoskeletal alterations observed after injury have complex relationships with strength imbalance and restricted muscle growth. Each of these contributes to osseous deformity and may interact together with limb disuse to induce glenohumeral deformities following BPBI. While strength imbalance, restricted muscle growth, and disuse all play a crucial role in deformity development and progression, which combinations of these contribute specifically to postganglionic or preganglionic injury sequelae remain unclear.

## 5 Summary and Future Directions

BPBI causes lifelong arm impairment in 30–40% of those affected [2], resulting in muscle weakness [93], osseous deformity [25], and postural deformity [94]. Injuries can occur from either a nerve rupture or nerve avulsion. Nerve rupture presents with shoulder contracture and limb disuse and is mimicked using postganglionic neurectomy murine models [69, 72]. Nerve avulsion presents with limb disuse without contracture and is mimicked with preganglionic neurectomy murine models [71, 74]. Morphological deformities develop in both the humerus and scapula but are more severe in the scapula following postganglionic injury [32, 67]. Osseous changes differ between postganglionic and preganglionic injury, with glenoid deformity following postganglionic injury and humeral growth deficits resulting in a smaller humeral head following preganglionic injury [67, 77]. Muscle deficits are greater following preganglionic injury than postganglionic injury for muscle length, mass, and sarcomere length [74]. In humans, the greatest muscle deficits occur in the subscapularis muscle [35, 47, 50].

Strength imbalance, restricted muscle growth, and limb disuse have all been implicated as factors contributing to deformity development following BPBI, but individual contributions to each injury type and additional underlying mechanisms contributing to these factors remain unclear. While the development of macrostructural bone deformities and muscle detriments following BPBI is well established, clinical reports generally include only postganglionic injury or do not specify injury type (Table 2). More knowledge is needed about the differential effects of nerve rupture and nerve avulsion on musculoskeletal development in the shoulder. Determining how BPBI induces microstructural, tissue‐level, and metabolic changes in both bone and muscle, as well as their interactions, will lend insight into potential mechanisms behind deformity. Deepening our understanding of the primary contributing factors in the development and progression of BPBI will ultimately inform more effective treatments tailored towards each injury type.

**Table 2:** Summary table of reported/known metrics following BPBI.

	Range of motion	Joint contracture	Limb disuse	Macro‐level bony deformities		Micro‐level bony detriments		Muscle detriments (*muscle/injury dependent*)
				Scapula	Humerus	Scapula	Humerus	
Unspecified BPBI	Not reported	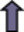	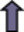	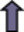	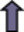	Not reported	Not reported	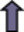
Nerve rupture (*postganglionic injury*)	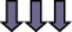	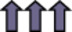	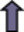	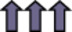	Inconclusive	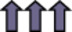	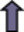	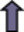
Nerve avulsion (*preganglionic injury*)	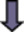	Not reported	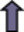	Not reported	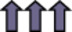	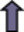	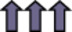	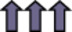
Observed in which studies?	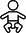 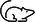	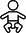 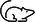	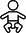 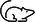	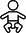 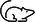	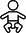 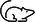	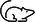	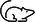	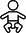 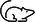

*Note:* 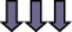 = substantially impacted 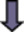 = impacted (arrow indicates direction of effect) 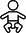 = human studies 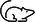 = rodent studies

## Supporting information

Grahn permission for figure 1.

required pages for using grahn figure.
