# Supplementary material for: Changes in Glenohumeral Musculoskeletal Development Following Brachial Plexus Birth Injury
Source: J Orthop Res. 2025 Jun 8;43(8):1367–77. doi: 10.1002/jor.26104 (PMC12258132; doi:10.1002/jor.26104)
Supplement: Supplementary file 1 — Grahn permission for figure 1. [file JOR-43-1367-s001.pdf]

NC STATE

Kyla Bosh <kbosh@ncsu.edu>

Request for Permission to Use Figure from Thesis

Grahn Petra <petra.grahn@hus.fi> Thu, Jun 6, 2024 at 11:27 AM
To: Kyla Bosh <kbosh@ncsu.edu>

Hi Kyla,

Yes, you may use the image as long as you reference the original source in your review paper. I can send you the original JPEG and PDF versions if you need them. Good luck with your paper!

BR, Petra Grahn

Petra Grahn

Hand surgeon

HUS Helsinki University Hospital, New Children´s Hospital

Department of pediatric orthopedics and traumatology

FI-00029 HUS | Stenbäckinkatu 9, Helsinki, Finland

HUS

Från: Kyla Bosh <kbosh@ncsu.edu>
Skickat: torsdag 6 juni 2024 18:06
Till: Grahn Petra <petra.grahn@hus.fi>
Ämne: Request for Permission to Use Figure from Thesis

Du får inte e-post ofta från kbosh@ncsu.edu. Se varför det här är viktigt.

[Quoted text hidden]

Huomaathan, että viesti on tullut HUSin ulkopuoliselta lähettäjältä. Avaa mahdolliset linkit ja liitteet vain, jos olet varma lähettäjästä.
