# Supplementary material for: Changes in Glenohumeral Musculoskeletal Development Following Brachial Plexus Birth Injury
Source: J Orthop Res. 2025 Jun 8;43(8):1367–77. doi: 10.1002/jor.26104 (PMC12258132; doi:10.1002/jor.26104)
Supplement: Supplementary file 2 — required pages for using grahn figure. [file JOR-43-1367-s002.pdf]

ResearchGate

See discussions, stats, and author profiles for this publication at: https://www.researchgate.net/publication/355437409

Improving Shoulder Function in Brachial Plexus Birth Injury

Thesis · August 2021

DOI: 10.13140/RG.2.2.14949.52967

CITATIONS
0

READS
11,156

1 author:

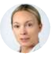

Petra Grahn
Helsinki University Central Hospital
38 PUBLICATIONS **94** CITATIONS

SEE PROFILE

All content following this page was uploaded by Petra Grahn on 20 October 2021.

The user has requested enhancement of the downloaded file.

Department of Pediatric Orthopedics and Traumatology
Helsinki University Hospital
and
Faculty of Medicine
Doctoral Programme in Clinical Research
University of Helsinki
Finland

IMPROVING SHOULDER FUNCTION IN BRACHIAL PLEXUS BIRTH INJURY

PETRA GRAHN-SHAHAR

DOCTORAL DISSERTATION

To be presented for public discussion with the permission of
the Faculty of Medicine of the University of Helsinki,
in Niilo Hallman's auditorium, HUS Parksjukhuset,
on August 27th 2021, at 13 noon.

Helsinki 2021

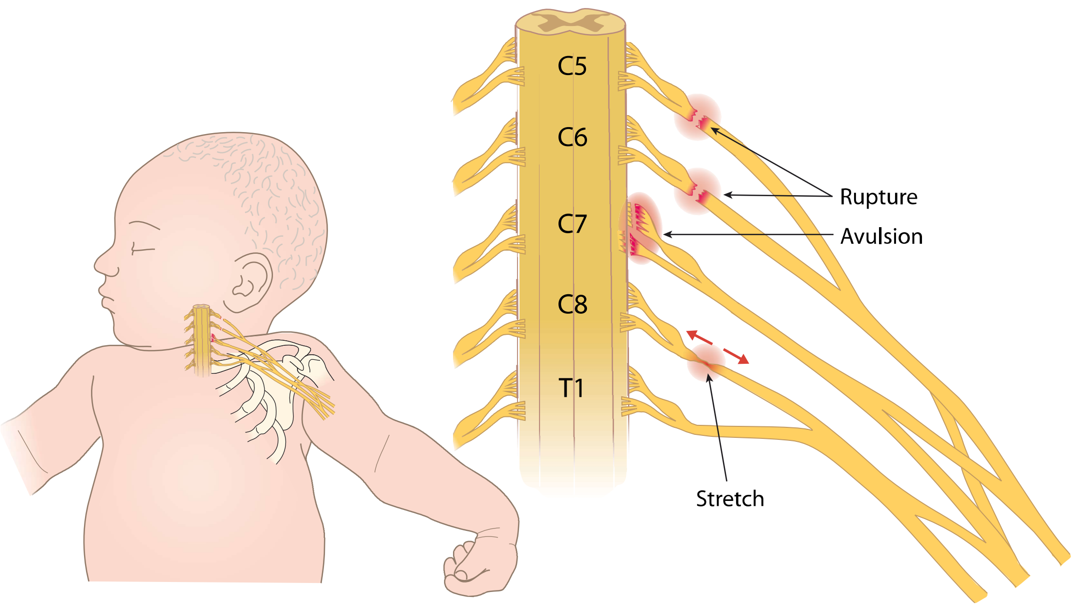

Figure 2 Child with complete plexus injury

Child showing typical “waiters tip” position with extended elbow, and internally rotated flexed wrist. C5-C6 roots ruptured, C7 avulsed from the spinal cord, C8 stretched.
